# Comparison of experimental peri-implantitis models after application of ex vivo BMP2 gene therapy using periodontal ligament stem cells

**DOI:** 10.1038/s41598-020-60341-7

**Published:** 2020-02-27

**Authors:** Shin-Young Park, Kyoung-Hwa Kim, Sungtae Kim, Sang-Hoon Rhee, In-Sung Yeo, Seong-Joo Heo, Yong-Moo Lee, Yang-Jo Seol

**Affiliations:** 1 Department of Periodontology and Dental Research Institute, School of Dentistry, Seoul National University, Seoul, 03080 Korea; 2 Department of Dentistry and Dental Research Institute, School of Dentistry, Seoul National University, Seoul, 03080 Korea; 3 Department of Dental Biomaterials Science, School of Dentistry, Seoul National University, Seoul, 03080 Korea; 4 Department of Prosthodontics and Dental Research Institute, School of Dentistry, Seoul National University, Seoul, 03080 Korea

**Keywords:** Experimental models of disease, Stem-cell research

## Abstract

Peri-implantitis is an inflammatory disease that results in bone destruction around dental implants. A preclinical study using beagle models is frequently performed prior to clinical application in dentistry. Previously, we proposed an immediate peri-implantitis experimental model with a shorter experimental duration and less expense than the conventional experimental model. However, the differences in the regenerative outcomes between the immediate and conventional models were not fully revealed. In this study, we aimed to compare the regenerative outcomes between both models when ex vivo BMP2 gene therapy using autologous periodontal ligament stem cells (B2/PDLSCs) was applied to peri-implantitis defects. The results showed that the defect depths were significantly different between both models. New bone formation occurred in both models, but there were significant differences between the models. More than 70% of the defects were filled with newly formed bone in the conventional model, whereas 30–40% of the defects were filled in the immediate model. However, after adjustment for the differences in the defect depths between the models, the statistically significant differences in the regenerative outcomes between the models were lost. In conclusion, the inferior regenerative outcome of an immediate peri-implantitis model at B2/PDLSCs transplantation resulted from the defect depths, not the model itself.

## Introduction

Peri-implantitis is an inflammatory disease induced by pathogenic bacterial accumulation on the implant surface^1,2^. Pathogenic bacteria initiate immune and inflammatory responses in the tissues that defend against the bacteria and sequester the damaged tissues for repair^3^. On this occasion, chemokines and cytokines secreted by inflammation around the implant stimulate the differentiation of monocytes into osteoclasts, leading to bone loss around the implant^4^.

However, due to the anatomical features of dental implants, such as the lack of periodontal ligaments (PDL), which are sources of stem cells and blood supply, bone loss resulting from peri-implantitis shows a pattern of extensive destruction and rapid progression compared to periodontal disease (gum disease), although periodontal disease and peri-implantitis have similar microorganism profiles^5,6^. As a result, when dental implants are involved in peri-implantitis, the longevity of the implants is severely jeopardized.

The anatomical differences of dental implants to natural teeth also influence the regenerative outcomes after treatment^6^. In animal and human studies, only limited amounts of bone regeneration has been reported above the defect at conventional bone regenerative procedures using bone substitutes and barrier membranes to cover the bone substitutes^7^. Therefore, innovative attempts to enhance the regenerative outcome in peri-implantitis defects are ongoing issues in dentistry.

**Figure 1:**
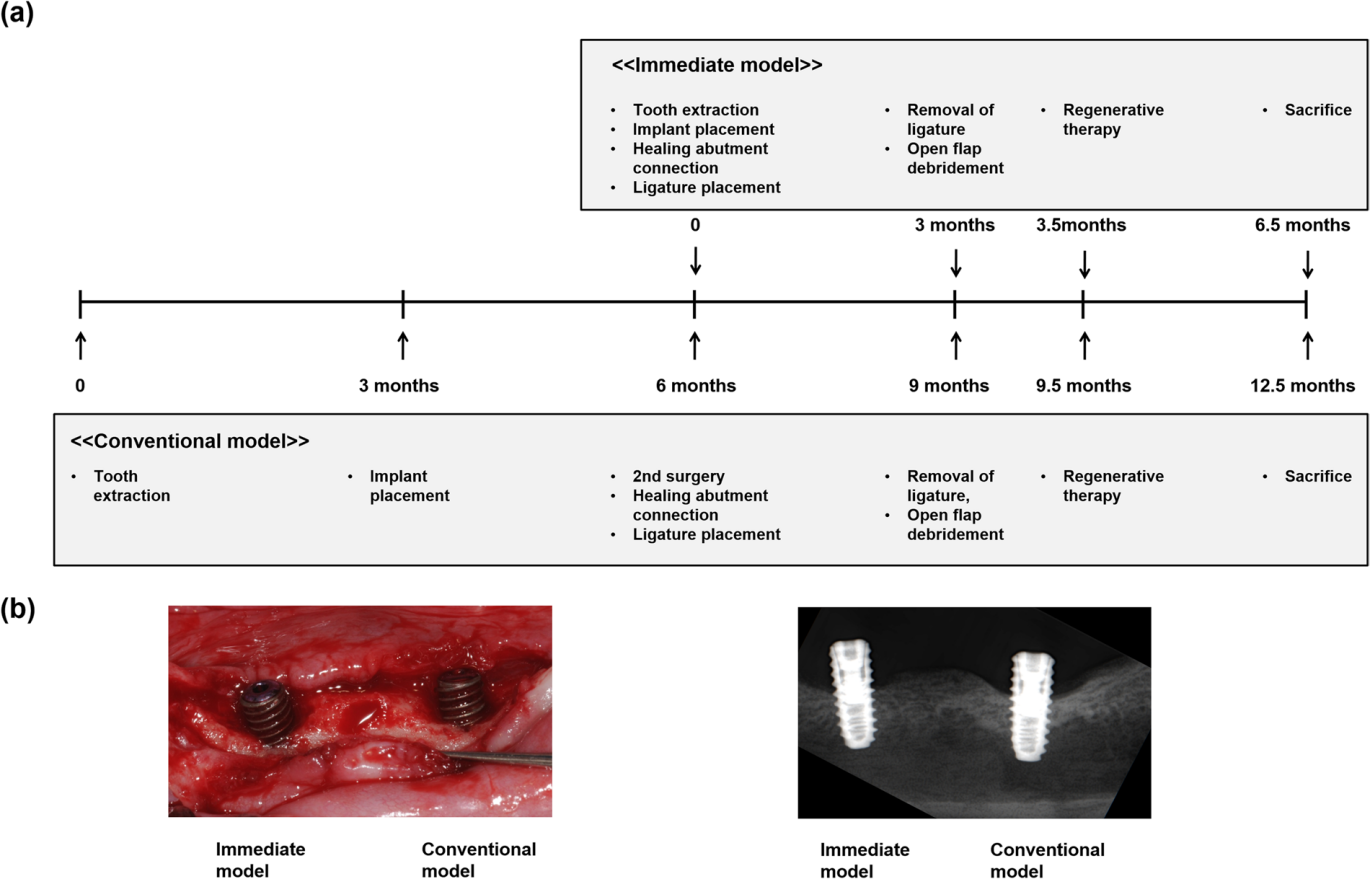
Summary of the experimental models. (**a**) The experimental flowchart, (**b**) the clinical and radiographic photos of the defects at the time of open flap surgery (left, immediate model; right, conventional model).

Nevertheless, the most challenging obstacles to developing an innovative treatment modality are the experimental models. The most widely applied model, which was first proposed by Lindhe *et al*. and modified by Berglundhe *et al*. to study the pathology of peri-implantitis, requires at least 10 months to make peri-implantitis defects to test, including 3 months of healing for the socket after tooth removal, 3 months of healing after dental implant placement, and more than 4 months of allowance for bacterial accumulation to induce peri-implantitis^5,8–12^. For experiments about regenerative therapy, it takes at least 13 months including more than 10 months of peri-implantitis induction and more than 3 months of healing after treatment to yield results. (Figure 1a) Therefore, the conventional model is too time-consuming and expensive to validate new therapies that are at risk of failure although this model shows a similar etiology and defect morphology with naturally occurring human peri-implantitis defects.

Previously, we proposed an immediate peri-implantitis model as a pilot test model for peri-implantitis treatment^13^. In this model, there is no need for a healing period after tooth removal and the placement of dental implants. Similar to human clinical procedures for immediate dental implant placement, a dental implant is immediately placed into a fresh extraction socket, and a ligature is placed around the healing abutment to allow plaque accumulation on the same day. After 3 months, a crater-like peri-implantitis defect manifests, and peri-implantitis treatment can be applied. However, our previous study showed that the defect was significantly deeper in the immediate model than in the conventional model. In addition, the progression of bone loss over 3 months was more rapid in the immediate model than in the conventional model. Finally, at the conventional regenerative procedures with bone substitutes and barrier membrane, the regenerative outcomes were significantly different between the models, although the immediate model had obvious advantages related to the experimental period and expenses .

In our previous study, we tested bone morphogenetic protein-2 (BMP-2)-producing PDL stem cells (PDLSCs) for the reconstruction of peri-implantitis defects^14^. As mentioned above, PDLs are a source of mesenchymal stem cells (MSCs) used in bone remodelling in the oral cavity. We obtained PDL stem cells from the removed natural teeth of dogs, which were transfected by adenoviral vectors carrying the human BMP-2 gene (B2/PDLSCs). As a result, B2/PDLSCs secreted BMP-2 at a low concentration for 3 weeks and yielded successful results in bone regeneration. Comparing to conventional regenerative procedures with or without PDLSCs, the significant new bone formation was observed on the implant surface previously contaminated by pathogenic bacteria (re-osseointegration), and more than 70% of the defects were filled with the newly formed bone. The most encouraging point of this study was the quantity and quality of the newly formed bone. Whereas previous studies showed that newly formed bone induced by the BMP-2 protein was immature and woven bone, B2/PDLSCs produced mature and lamellar new bone above the defects^15^. Therefore, it was concluded that B2/PDLSCs are potent bone inducers and could be used to regenerate peri-implantitis defects.

After confirming the differences of an immediate peri-implantitis model in regenerative outcome to conventional model, we wondered whether the inferiority of the immediate peri-implantitis model could be overcome by potent bone inducers such as B2/PDLSCs. Thus, we developed peri-implantitis defects with each model, transplanted B2/PDLSCs into the defects and compared the regenerative outcomes of both models.

## Results

During the entire experiment, none of the implants failed in either the immediate or conventional model. The shapes of the peri-implantitis defects at the time of B2/PDLSC transplantation were wide and crater-like in both models, but the defect depths were deeper and the implants were more exposed in the immediate model (Fig. 1b). More bone loss was observed on the buccal side than on the lingual side in both models. After transplantation surgery, the surgery sites had healed well regardless of the experimental model, and there were no adverse complications.

### Histological and histomorphometric analyses

**Figure 2:**
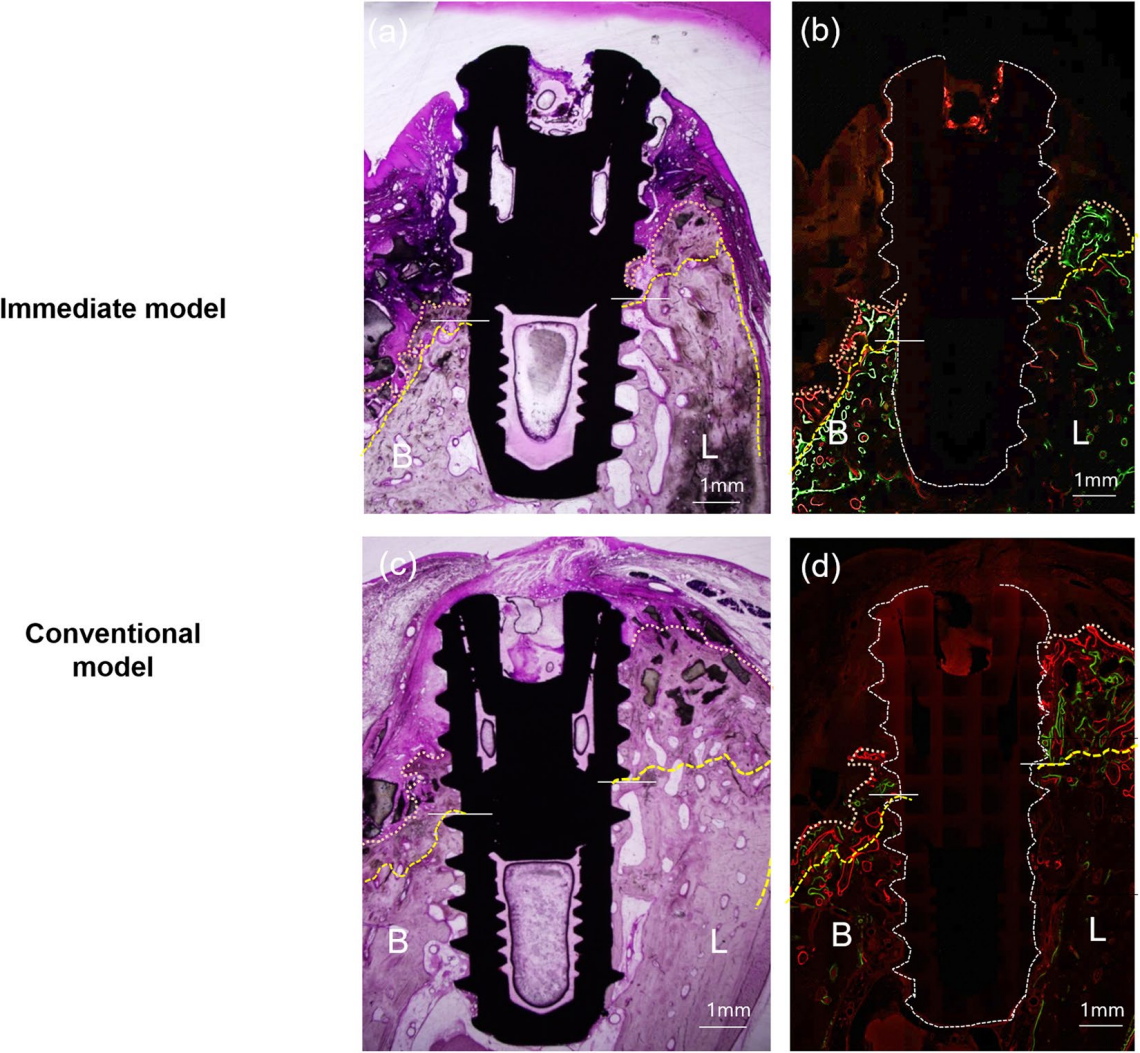
Representative microscopic and confocal laser scanning microscopic photographs of histological sections. In both models, B2/PDLSCs produced significant new bone formation on both sides, but the amount of new bone was greater in the conventional model (**c**,**d**) than in the immediate model (**a**,**b**). Multiple stains on undecalcified ground sections (**a**,**b**), 1.5X original magnification (**a**–**d**); bone labelling at 4 weeks (green; oxytetracycline HCl) and 8 weeks (orange; xylenol orange). yellow line, bottom of the defect; white line, dental implant; pink line, outline of newly formed bone. B, buccal; L, lingual; bars indicate the bottom of the defect on either side.

**Figure 3:**
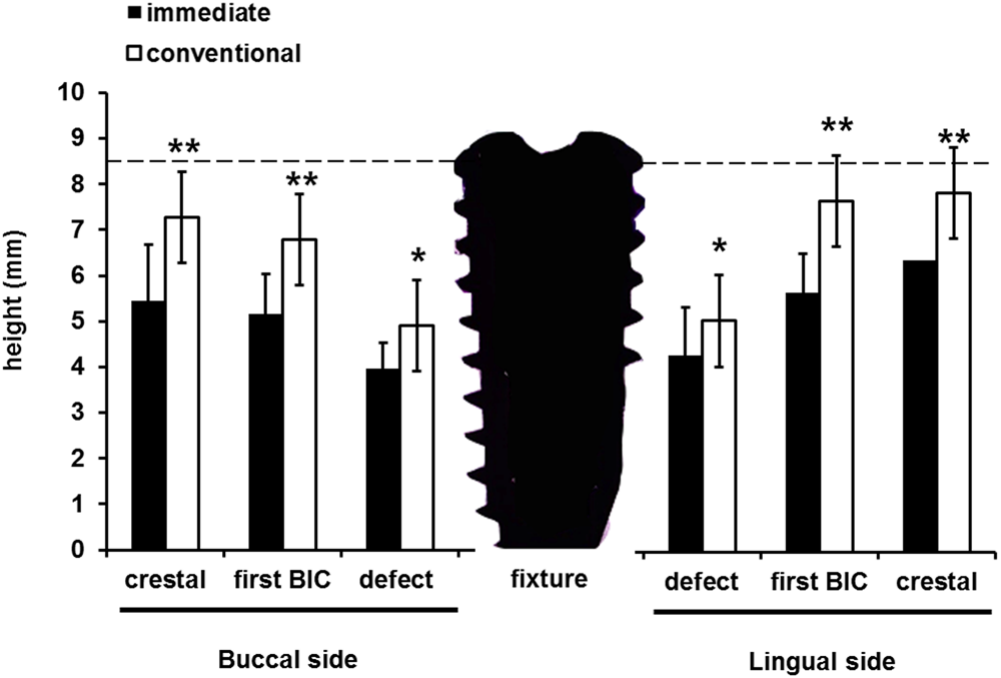
Histomorphometric analysis. Significant differences were observed between the immediate and conventional models in defect depth, first BIC height, and crestal height. *Indicates statistical significance at p $<$ 0.05 **indicates statistical significance at p $<$ 0.01.

**Figure 4:**
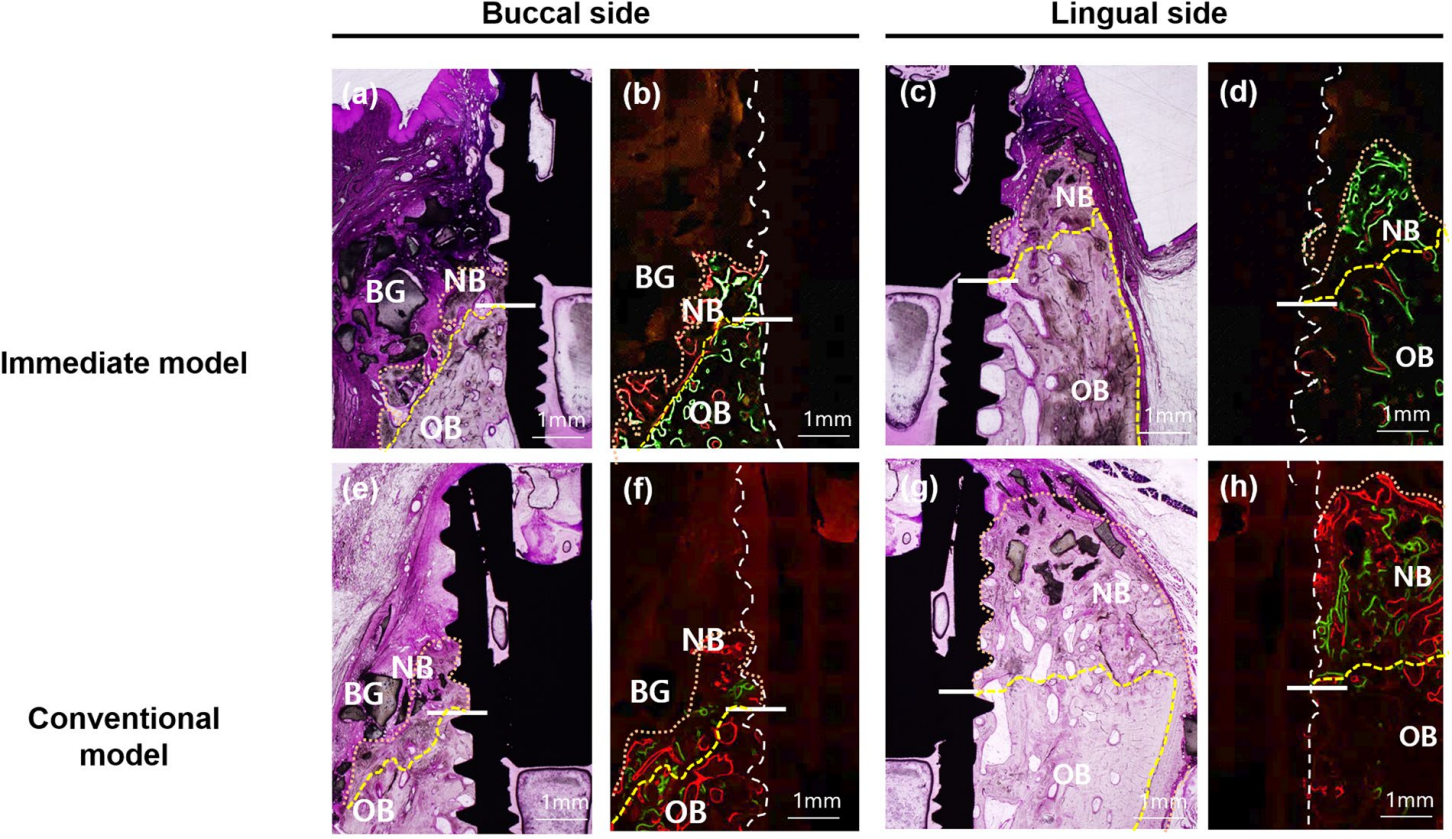
Magnification of the buccal side in the immediate model (**a**–**d**) and the conventional model (**e**–**h**). The amount of newly formed bone above the defect in the buccal side was similar regardless of the peri-implantitis model. However, the new bone on the buccal side was greater in the conventional model than in the immediate model. Multiple stains on undecalcified ground sections (**a**,**c**), 2X original magnification (**a**–**d**); bone labelling at 4 weeks (green; oxytetracycline HCl) and 8 weeks (orange; xylenol orange). Yellow line, bottom of the defect; white line, dental implant; pink line, outline of newly formed bone. B; buccal, L; lingual, BG; bone graft, NB; new bone, OB; old bone; bars indicate the bottom of the defect.

Representative histological images are shown in Fig. 2, and histomorphometric measurements, which were made in these images to compare the quantitative differences between the models, are summarized in Fig. 3. As previously mentioned, the defects were deeply formed in the immediate model, and the defect height (from the apex of the implant to the bottom of the defect) was smaller in the immediate model than in the conventional model on both the buccal and lingual sides. The most coronal level of the direct contact of bone to implant (first BIC) height from the apex was also significantly smaller in the immediate model on both sides. The first BIC height is a meaningful index for indicating functional bone supports that can bear bite forces during mastication. The crestal height from the apex was also smaller in the immediate model than in the conventional model. In magnified images (Fig. 4), newly formed bone can be observed in both models. In the immediate model, new bone formation above the defects was observed, but the amount of new bone was greater on the lingual side than on the buccal side. Approximately 1 thread of implant height was covered by newly formed bone on the buccal side, whereas 1.5 threads were covered by new bone on the lingual side. In the conventional model, the new bone formation above the defects was greater than in the immediate model on both sides. In particular, on the lingual side of the conventional model, newly formed bone was almost at the level of the implant shoulder, and less than 1 thread of dental implant surface was not in direct contact with the bone. The histomorphometric measurements of defect depth, amount of new bone formation and re-osseointegration are summarized in Table 1. The defect depths were more than 4 mm in the immediate model and approximately 3.5 mm in the conventional model. The newly formed bone area was significantly greater in the conventional model than in the immediate model on the buccal side $(p=0.010)$ and almost significantly greater on the lingual side $(p=0.080)$. The total mineralized bone area was significantly different between models. The re-osseointegration height (vertical height from the bottom of the defect (BD) to the first BIC level) was approximately 1.5 mm in the immediate model and more than 2 mm in the conventional model and was significantly different on both sides. Regarding the vertical bone fill (the ratio of the re-osseointegration height to the defect depth), approximately 30% of the defects were re-osseointegrated in the immediate model, whereas more than 60% were re-osseointegrated in the conventional model ($p=0.016$ on the buccal side; $p=0.017$ on the lingual side).

**Table 1:** Histological measurements in the immediate and conventional models. Statistical analyses were performed with Student’s t-test. The statistical significance level was p $<$ 0.05. *Bone graft area directly contacting the bone. ^†^The re-osseointegration height was measured from the bottom of the defect to the first BIC. ^‡^Vertical bone fill (percentage) was calculated as the ratio of the re-osseointegration height to the defect depth.

Side	Measurements	Immediate	Conventional	Difference	p-value
Buccal side	Defect depth	4.5 (0.6)	3.6 (0.8)	0.9	0.023
	Newly formed bone area (mm^2^)	0.9 (0.5)	4.4 (3.1)	−3.5	0.010
	Area of the bone graft (mm^2^)*	0.2 (0.2)	1.6 (1.9)	−1.4	0.081
	Total mineralized tissue area (mm^2^)	1.1 (0.7)	6 (4.6)	−4.9	0.016
	Re-osseointegration height (mm)^†^	1.3 (0.7)	2 (0.4)	−0.7	0.031
	Vertical bone fill (percentage)^‡^	29.0 (16.4)	60.0 (25.1)	−31.0	0.016
Lingual side	Defect depth (mm)	4.2 (0.8)	3.5 (0.3)	0.8	0.031
	Newly formed bone area (mm^2^)	2.1 (1.1)	3.6 (1.7)	−1.5	0.080
	Area of bone graft (mm^2^)	0.2 (0.2)	0.9 (0.7)	−0.7	0.021
	Total mineralized tissue area (mm^2^)	2.4 (1.1)	4.5 (2.2)	−2.1	0.042
	Re-osseointegration height (mm)^†^	1.6 (0.9)	2.4 (0.5)	−0.8	0.031
	Vertical bone fill (percentage)^‡^	39.6 (26.1)	70.9 (15.9)	−31.3	0.017

### Effects of the peri-implantitis models on bone regeneration

According to the histomorphometric measurements, there were significant differences in the regenerative outcomes between the models. However, because regenerative outcomes are significantly influenced by defect morphology, it could not be determined whether the differences in bone regeneration between the models were due to the experimental models themselves. Therefore, we performed a multilevel mixed-effects regression analysis after adjusting for defect depth (Table 2).

**Table 2:** Multilevel mixed-effects regression analysis evaluating the effect of the peri-implantitis model on the regenerative outcome of BMP2 gene therapy. A mixed-effects regression analysis was performed, and the significance level was set at p $<$ 0.05. Adjusted model 1: random-effects – dog. Adjusted model 2: fixed-effects – defect depth, dog *Bone graft area directly contacting the bone.

	Crude model	p-value	Adjusted model 1	p-value	Adjusted model 2	p-value
	Coefficient (CI)		Coefficient (CI)		Coefficient (CI)	
Buccal side						
Newly formed bone area (mm^2^)	0.11 (0.03–0.19)	0.010	0.11 (0.04–0.18)	0.001	0.08 (–0.01–0.17)	0.079
Area of the bone graft (mm^2^)	0.15 (–0.02–0.32)	0.081	0.15 (0.00–0.30)	0.044	0.07 (–0.09–0.23)	0.397
Total mineralized tissue area (mm^2^)	0.07 (0.02–0.13)	0.016	0.07 (0.02–0.12)	0.003	0.05 (–0.01–0.11)	0.129
Re-osseointegration height (mm)	0.41 (0.04–0.78)	0.031	0.41 (0.10–0.73)	0.011	0.26 (–0.08–0.60)	0.140
Vertical bone fill (percentage)	1.13 (0.24–2.01)	0.016	1.13 (0.37–1.88)	0.003	0.75 (–0.60–2.10)	0.278
Lingual side						
Newly formed bone area (mm^2^)	0.14 (–0.02–0.30)	0.080	0.14 (0.00–0.28)	0.044	0.1 (–0.03–0.22)	0.127
Area of the bone graft (mm^2^)	0.48 (0.08–0.87)	0.021	0.48 (0.14–0.82)	0.005	0.36 (0.03–0.69)	0.033
Total mineralized tissue area (mm^2^)	0.12 (0.01–0.24)	0.042	0.12 (0.02–0.23)	0.017	0.09 (–0.01–0.19)	0.069
Re-osseointegration height (mm)	0.30 (0.00–0.60)	0.050	0.30 (0.04–0.56)	0.022	0.23 (0.00–0.47)	0.050
Vertical bone fill (percentage)	1.00 (0.18–1.82)	0.021	1.00 (0.29–1.70)	0.005	0.69 (–0.08–1.47)	0.080

On the buccal side, the type of experimental model significantly influenced all the measurements except the area of the bone graft. After adjusting for individual differences in the animals, the peri-implantitis model still significantly affected bone regeneration in all measurements. However, after adjusting for defect depth and animals, the type of peri-implantitis model did not significantly affect bone regeneration, re-osseointegration, or vertical bone fill.

The lingual side showed a similar pattern to the buccal side. The type of experimental model was a significant factor in the area of the bone graft, the total mineralized area, and vertical bone fill. After adjusting for animals, all the measurements were significantly affected by the experimental model. However, after adjusting for animals and defect depth, the peri-implantitis model only significantly influenced the area of the bone graft, although the effects on total mineralized tissue area, re-osseointegration height and vertical bone fill approached statistical significance.

## Discussion

In this study, we tested an immediate peri-implantitis model and a conventional peri-implantitis model when a potent bone-inducing morphogene, BMP-2, was applied with a method of ex vivo gene delivery using PDLSCs.

PDLSCs are dental originated MSCs that are isolated from root surfaces of an extracted tooth. Among MSCs, bone marrow stromal cells (BMSCs) have long been used for bone engineering^16^. PDLSCs are the progenitor cells of periodontal tissues, such as bone, cementum, gingiva and PDL, which are used in the construction of functional units of teeth and supporting tissues. Previously, we tested BMSCs and PDLSCs in a surgically created peri-implant defect model of beagle, PDLSCs yielded comparable results in bone regeneration to BMSCs^17^. In ex vivo BMP2 gene delivery, both cell type were effective to induce a significant amounts of bone regeneration although it was performed in the different experimental models^18,19^. Ex vivo BMP2 gene delivered BMSCs were applied into rat critical-sized calvarial defects, whereas B2/PDLSC were transplanted into the canine peri-implantitis model. Due to the accessibility of PDLSCs without additional intervention for harvesting MSCs in case of the removal of teeth, PDLSCs were satisfactory options for ex vivo BMP2 gene delivery in the oral cavity.

The regenerative outcomes of the cells or interventions are also dependent on the experimental model. When PDLSCs alone were transplanted into a peri-implantitis defects in our previous study, PDLSCs did not produce significant bone regeneration compared to the control group^14^. However, another previous study using a surgically created peri-implant defect that is a fresh wound site without bacterial contamination showed that transplantation of PDLSCs alone was effective in bone regeneration around the dental implant. A peri-implantitis defect results from bone loss due to inflammatory responses related to bacterial deposition on dental implant surfaces. It is a more challenging model than a surgically created defect requiring more osteogenic potential to induce bone regeneration on previously contaminated surfaces.

To improve bone regeneration, a tissue engineering-based strategy using stem cells, signaling molecules and scaffolds has been attempted and has produced successful results^20–22^. In this context, we established effective bone regeneration procedures using B2/PDLSCs with bone substitutes for peri-implantitis defects in previous study^14^. In bone engineering, adenoviral vectors or adenovirus-associated viral vectors (AAVs) are most commonly used for BMP2 gene delivery due to their patterns of release of BMP-2^23^. Unlike retroviral vectors, target genes carried by adenoviral vectors are not inserted into the host cell genome but are maintained within cells as episomes and are produced only for short periods. Due to prolonged secretion at a low concentration, BMP2 gene therapy produced significant new bone formation above the peri-implantitis defects, and greater than 70% vertical bone fill was observed; rhBMP-2 therapy for peri-implantitis defects resulted in approximately 30% vertical bone fill^24^. Xu *et al*. also investigated the effect of ex vivo BMP2 gene therapy using adipose-derived stem cells (B2/ASCs) in a peri-implantitis model^25^. B2/ASC transplantation produced significant amounts of new bone above the defects, which was similar to our study results with the conventional model. However, as ex vivo gene delivery using stem cells is very expensive and complicated due to the cell expansion process, gene-activated matrices such as adipose tissues or muscles are recruited as gene delivery carriers. Recently, Virk *et al*. proposed “same day” ex vivo gene delivery for bone repair with buffy coat cells from bone marrow transfected by lentiviral vectors carrying the BMP2 gene.^26^

Although technology advances day by day, experimental models for investigating peri-implantitis treatment were still time-consuming and require more than 13 months to confirm the effects of the therapy. From this point of view, we designed an immediate peri-implantitis model as a pilot model to screen the regenerative outcome over a short period. However, we failed to demonstrate the equivalence between the immediate and conventional models in defect morphology as well as regenerative outcome under conventional bone regeneration procedures with bone substitutes alone^13^. Peri-implantitis defects induced by both models showed similar shapes, but rapid bone loss and deeper defect depths were observed in the immediate model. The significant differences in bone regeneration, such as in re-osseointegration and vertical bone fill, between the two models remained significant after adjusting for defect depths and differences in individual dogs.In addition, the increased surface exposure in the immediate model is also disadvantageous in terms of re-osseointegration. During the early phase of healing, coronal portions of implant surfaces are orally exposed and contaminated by bacterial deposits, which are also unfavourable for re-osseointegration. In the study by Alhag *et al*.^27^, when supracrestally exposed implants were removed and placed into fresh sockets, the contaminated coronal portion exhibited less than 50% BIC, whereas the apical portion in which osseointegration normally occurs showed greater than 80% BIC. Therefore, regeneration in peri-implantitis defects induced by the immediate model is more challenging than in those induced by the conventional model.

Nevertheless, B2/PDLSCs transplantation overcame the inferiority in bone regeneration of the immediate model seen in our previous study. Therefore, the immediate peri-implantitis model is sensitive at the regenerative potential of the biomaterials or intervention and useful indicator to evaluate the regenerative potential of new materials as a pilot test model. However, a well-designed, full-scale preclinical test using a conventional peri-implantitis model is required to confirm the regenerative outcomes before clinical application.

In conclusion, the regenerative effect of B2/PDLSCs was consistently observed in both models, although less regenerated bone was observed in the immediate model compared to the conventional model. However, the differences between models resulted from the differences of defect depths between models. A preliminary study using an immediate model with a new substance can provide important information for determining whether to transition to a full-scale preclinical regenerative study.

## Methods

### Animals

In this study, a total of four beagles (weighing 10 to 12 kg) were used. All animals had fully erupted permanent dentition. This study complied with the Animal Research: Reporting of In Vivo Experiments (ARRIVE) guidelines for preclinical studies. This study protocol was approved by the Institute of Laboratory Animal Resources, Seoul National University (SNU-120908-5).

### Peri-implantitis model induction

All surgical procedures were conducted under general anaesthesia using xylazine hydrochloride (Rompun, Bayer Korea, Seoul, Korea) and ketamine hydrochloride/zolazepam hydrochloride (Ketamine HCl, Yuhan, Seoul, Korea) and under local anaesthesia using lidocaine hydrochloride with 1:100,000 epinephrine (Lignospan, Septodont, Cedex, France). The experimental flowchart is summarized in Fig. 1. Induction of the experimental peri-implantitis model was described in our previous study^14^. The experimental design involved peri-implantitis defects induced by the conventional model in the 4th premolar (P4) region and by immediate injection in the 3rd premolar (P3) region.

Briefly, the mandibular P4 and first molars (M1) of each animal were extracted, and the wounds were allowed to heal. After 3 months, dental implants with a diameter of 3.5 mm and a length of 8.5 mm (TSIII SA fixture [sand-blasted with alumina and acid-etched with Ra 2.5–3.0 micrometre], Osstem, Seoul, Korea) were installed and submerged in the P4 region. After 3 months of healing, uncovering surgery of the dental implants in the P4 region was performed, and healing abutments were connected to the dental implants. On the same day, the mandibular third premolars (P3) were extracted, and dental implants were immediately placed into the extraction sockets. The healing abutments were directly connected to the implants.

To allow bacterial deposition around the dental implants, ligatures (cotton braided retraction cord, Ultrapak, Ultradent Products, Inc., South Jordan, UT, USA) were wound below the healing abutments of both implants in the P3 and P4 regions. Additional ligatures were applied below the pre-existing ligatures every fourth week for 3 months. After peri-implantitis defects began to manifest, the ligatures were removed. For further regenerative therapy, the inflammatory tissues and contaminated implant surfaces were thoroughly cleaned with open flap debridement and a dental water jet (Aqua-pik®, Aquapik Co., Seoul, Korea) at a high-pressure setting (1200 pulses per minute).

### Preparation of autologous canine PDLSCs

Autologous canine PDLSCs were obtained from the extracted mandibular P4 and M1 cells. The PDL was gently separated from the middle third of the roots, and PDLSCs were cultured per the technique described by Seo *et al*.^28^.

### Preparation of autologous B2/PDLSCs

In this study, ex vivo adenoviral BMP2 (AdBMP2) gene delivery of PDLSCs was performed. The construction of AdBMP2 has been described previously^29^. Eighteen hours before surgery, PDLSCs from passages two through three at a density of 1 $\times$ $1{0}^{6}$ cells were plated onto a 100-mL dish and transduced with AdBMP2 at a multiplicity of infection (MOI) of 100 pfu for 4 hours. On the day of surgery, after confirmation of the expression of BMP-2 by the B2/PDLSCs with ELISA, cells were detached using a sterile trypsin-EDTA solution and mixed with 200 mL of collagen hydrogel at a pH of 7.4 (1% purified porcine skin-derived type I atelo-collagen (Matrixen-PSPVR, Bioland, Seoul, Korea), 0.001 N HCl, 26 mM NaHCO3, 20 mM HEPES, and 0.025 N NaOH).

### B2/PDLSC transplantation into peri-implantitis defects

Two weeks after open flap debridement, B2/PDLSCs were prepared for transplanting into the defects. Regarding the surgical procedures, a full-thickness flap was elevated, and the flap, previously contaminated by bacterial accumulation, was thoroughly decontaminated using a dental water jet at a high-pressure setting and dental floss (Superfloss®, P and G, Cincinnati, OH, USA) soaked in chlorhexidine^30^. To stimulate bone healing, a decortication procedure that punctured the cortical bone to expose the bone marrow was performed with a high-speed round burr. After completion of the preparation, HA particles (ranging from 425 to 600 micrometres, calcium hydroxide:phosphoric acid = 10:6; synthesis of HA particles was described in detail by Park *et al*.^14^) mixed with collagen hydrogel containing B2/PDLSCs were transplanted into the peri-implantitis defects. To secure the graft materials, absorbable collagen membranes (Bio-gide®, Geistlich Biomaterials, Wolhusen, Switzerland) were placed onto the grafts and fixed with two fixation screws (Ti bone screw, Warantec, Seoul, Korea) on the buccal side. To stabilize the barrier membrane and graft materials, a horizontal matrix suture was applied over the membrane. Primary closure of the periosteal flap was performed using a releasing incision and a tension-free suture.

Intravenous injections of antibiotics (Cefazolin, 30 mg/kg, Chong Kun Dang Pharmaceutical Corp., Seoul, Korea) was performed for 3 days after surgery. The animals were fed a soft diet for 2 weeks. Oxytetracycline HCl (Sigma, St. Louis, MO, USA; 20 mg/kg) and xylenol orange (Sigma; 20 mg/kg) were administered after 4 and 8 weeks for fluorescent bone labelling. Three months after surgery, the animals were sacrificed, and samples were harvested.

### Histological examination and histomorphometric analysis

The samples were block-resected and fixed in 10 percentile neutralized buffered formalin. The samples were then dehydrated in a series of ethanol solutions of increasing concentration and embedded in media (Technovit 7200, Exakt, Hamburg, Germany). Undecalcified ground sections were prepared using a sawing and grinding technique^31^. The blocks were cut in a buccal-lingual plane parallel to the long axis of the implant. Specimens were ground and polished to a final thickness of approximately 50 micrometres using a cutting and grinding system (EXAKT Apparatebau, Norderstedt, Germany). Unstained slides were subjected to confocal laser scanning microscopy (CSLM) examination. Then, the slides were stained with a multiple stain solution (Polyscience, Inc., Warrington, PA, USA) for light microscopic examination. The slides were magnified using a light microscope (Olympus BH-2, Olympus Optical, Osaka, Japan), and images were captured with a digital camera for histomorphometric analysis.

**Figure 5:**
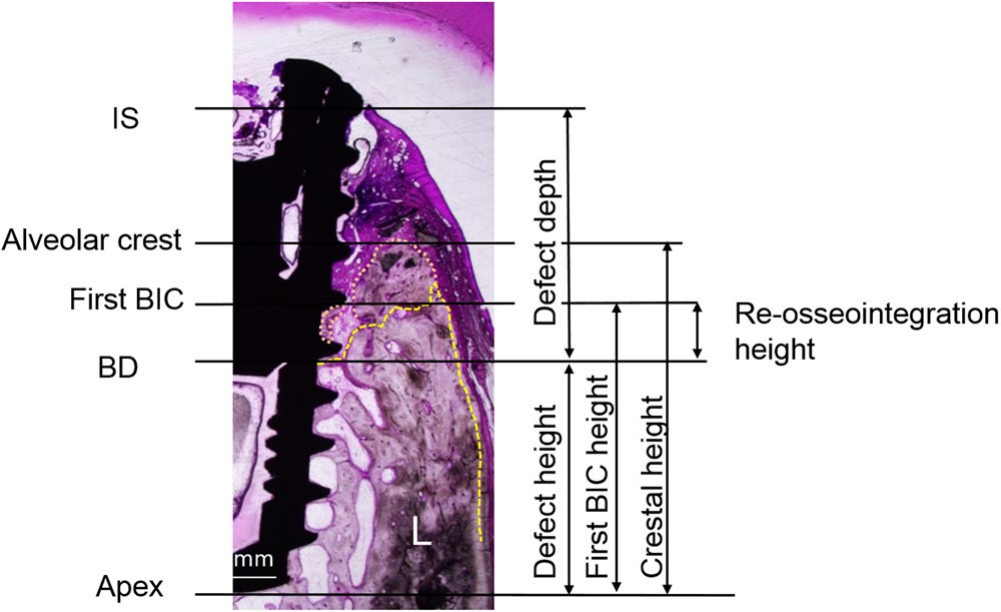
Histomorphometric landmarks in the histological images (original magnification, X 1.5): IS, implant shoulder; first BIC, the most coronal first bone-to-contact; BD, bottom of the defect; defect depth (mm), from the bottom of the defect to the implant shoulder; re-osseointegration height (mm), from the bottom of the defect to the first BIC; first BIC height (mm), from the implant apex to the first BIC; crestal height (mm), from the implant apex to the alveolar crest.

Histomorphometric analysis was carried out for the buccal and lingual sides of each implant as follows: (1) defect depth from the implant shoulder (IS) to the bottom of the defect (BD); (2) total mineralized tissue area (mm^2^) including the newly formed bone area and the area of the bone graft materials; (3) re-osseointegration height (mm), the vertical height from the BD to the most coronal first BIC level; (4) vertical bone fill (percentage), calculated as the ratio of the re-osseointegration height to the defect depth; (5) BIC within the re-osseointegrated bone (mm), a linear measurement of the bone in direct contact with the implant surface within the re-osseointegrated bone; (6) crestal height (mm), the vertical height from the apex of the implant to the most coronal level of the alveolar bone; and (7) the first BIC height (mm), the vertical height from the apex of the implant to the most coronal first C level of the BD (Fig. 5).

All measurements were performed using the computer software of an image analysis system (Tomoro Scope Eye 3.5 Image Analyzer; Techsan Digital Imaging, Seoul, Korea) by a blinded and experienced investigator (Kwak, Eun-Hye) who had not participated in any of the surgical procedures. The error between pre- and post-examination was less than 5%.

### Statistical analysis

Statistical analysis between the models was conducted using commercially available software (STATA/SE14 software, Stata Corp, College Station, TX, USA). The normality of the distribution of the measurements was tested with the Kolmogorov-Smirnov test. Student’s t-test was used to compare measurements between the models, and a multilevel mixed-effects regression analysis adjusting for dogs and defect depths was conducted to evaluate the effect of the model on the regenerative outcomes. Statistical significance was determined for p-values less than 0.05.

## Supplementary information

Supplementary Information.

## Data Availability

All data generated or analysed during this study are included in this published article (and its Supplementary Information files).
